# Liver Disease and Cell Therapy: Advances Made and Remaining Challenges

**DOI:** 10.1093/stmcls/sxad029

**Published:** 2023-04-13

**Authors:** Sheeba Khan, Sara Mahgoub, Nada Fallatah, Patricia F Lalor, Philip N Newsome

**Affiliations:** National Institute for Health Research, Biomedical Research Centre at University Hospitals Birmingham NHS Foundation Trust and the University of Birmingham, Birmingham, West Midlands, UK; Centre for Liver and Gastrointestinal Research, Institute of Immunology and Immunotherapy, University of Birmingham, Birmingham, West Midlands, UK; Liver Unit, University Hospitals Birmingham NHS Foundation Trust, Birmingham, Birmingham, West Midlands, UK; National Institute for Health Research, Biomedical Research Centre at University Hospitals Birmingham NHS Foundation Trust and the University of Birmingham, Birmingham, West Midlands, UK; Centre for Liver and Gastrointestinal Research, Institute of Immunology and Immunotherapy, University of Birmingham, Birmingham, West Midlands, UK; Liver Unit, University Hospitals Birmingham NHS Foundation Trust, Birmingham, Birmingham, West Midlands, UK; National Institute for Health Research, Biomedical Research Centre at University Hospitals Birmingham NHS Foundation Trust and the University of Birmingham, Birmingham, West Midlands, UK; Centre for Liver and Gastrointestinal Research, Institute of Immunology and Immunotherapy, University of Birmingham, Birmingham, West Midlands, UK; Department of Laboratory Medicine, Faculty of Applied Medical Sciences, Umm Al-Qura University, Makkah, Saudi Arabia; National Institute for Health Research, Biomedical Research Centre at University Hospitals Birmingham NHS Foundation Trust and the University of Birmingham, Birmingham, West Midlands, UK; Centre for Liver and Gastrointestinal Research, Institute of Immunology and Immunotherapy, University of Birmingham, Birmingham, West Midlands, UK; National Institute for Health Research, Biomedical Research Centre at University Hospitals Birmingham NHS Foundation Trust and the University of Birmingham, Birmingham, West Midlands, UK; Centre for Liver and Gastrointestinal Research, Institute of Immunology and Immunotherapy, University of Birmingham, Birmingham, West Midlands, UK; Liver Unit, University Hospitals Birmingham NHS Foundation Trust, Birmingham, Birmingham, West Midlands, UK

**Keywords:** chronic liver disease, mesenchymal stromal cells, extracellular vesicles, immunomodulation

## Abstract

The limited availability of organs for liver transplantation, the ultimate curative treatment for end stage liver disease, has resulted in a growing and unmet need for alternative therapies. Mesenchymal stromal cells (MSCs) with their broad ranging anti-inflammatory and immunomodulatory properties have therefore emerged as a promising therapeutic agent in treating inflammatory liver disease. Significant strides have been made in exploring their biological activity. Clinical application of MSC has shifted the paradigm from using their regenerative potential to one which harnesses their immunomodulatory properties. Reassuringly, MSCs have been extensively investigated for over 30 years with encouraging efficacy and safety data from translational and early phase clinical studies, but questions remain about their utility. Therefore, in this review, we examine the translational and clinical studies using MSCs in various liver diseases and their impact on dampening immune-mediated liver damage. Our key observations include progress made thus far with use of MSCs for clinical use, inconsistency in the literature to allow meaningful comparison between different studies and need for standardized protocols for MSC manufacture and administration. In addition, the emerging role of MSC-derived extracellular vesicles as an alternative to MSC has been reviewed. We have also highlighted some of the remaining clinical challenges that should be addressed before MSC can progress to be considered as therapy for patients with liver disease.

## Graphical Abstract

**Graphical Abstract:**
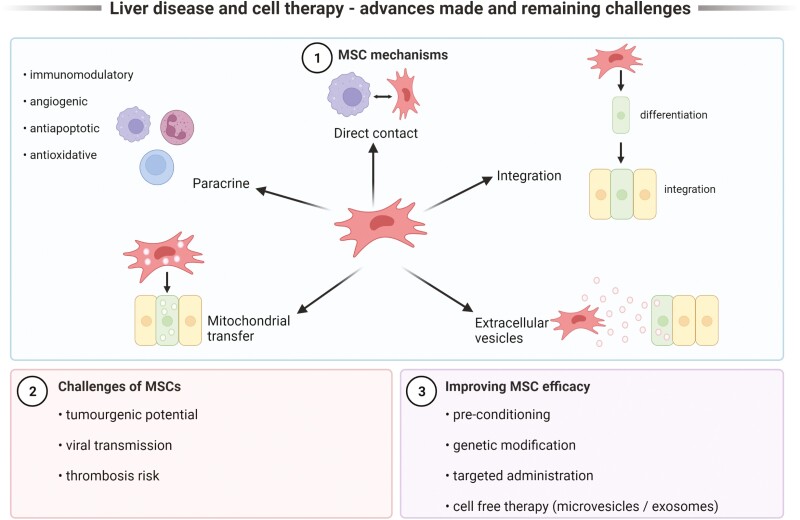

**Significance Statement**

There is extensive published literature that supports the safety and the efficacy of mesenchymal stromal cells (MSC) as a potential therapy for liver disease. It is the immunomodulatory and the anti-inflammatory role of MSC in the setting of inflammatory liver disease that has been the focus of the contemporary research. In this article, the authors have reviewed the key translational and the clinical studies with MSC in liver disease so far. The heterogeneity between different studies and the conflicting results have been highlighted. In addition, the emerging role of extracellular vesicles and the remaining challenges in the field have also been discussed.

## Introduction

The significant global disease burden associated with chronic liver disease,^1,2^ coupled with increasing mortality for patients on liver transplant waiting lists^3,4^ has fueled research efforts to seek alternative therapeutic agents. In particular, a wide range of cell therapies including regulatory T cells, hematopoietic stem cells, embryonic/pluripotent cells, and mesenchymal stromal cells (MSCs) have been studied in liver disease. These approaches all have their merits, but MSC in particular have been extensively studied due to their modest immunogenicity and capacity to modulate immune cells and inflammation for therapeutic benefit.^5^ There are currently 988 registered clinical trials studying MSCs search at www.clinicaltrials.gov) with at least 10 globally approved MSC therapies (Table 1).^6^

**Table 1.** Globally approved MSC therapies.

Name	MSC type	Indication	Country of approval/year
Alofisel	Human AT-MSC	Complex perianal fistula in Crohn’s disease	Europe (2018)
Prochymal (Remestemcel-L)	Human BM-MSC	Graft versus host disease	Canada (2012) New Zealand (2012)
Queencell	Human AT-MSC	Subcutaneous tissue defects	South Korea (2010)
Cupistem	Human AT-MSC	Crohn’s fistulae	South Korea (2012)
Cartistem	Human BM-MSC	Knee articular cartilage defects	South Korea (2012)
Stemirac	Human BM-MSC	Spinal cord injury	Japan (2018)
Stempeucel	Human BM-MSC	Critical limb ischemia	India (2016)
Cellgram-AMI	Human BM-MSC	Acute myocardial infarction	South Korea (2011)
Temcell HS inj	Human BM-MSC	Graft versus host disease	Japan (2015)
Neuronata-R	Human BM-MSC	Amyotrophic lateral sclerosis	South Korea (2014)

Abbreviations: MSC, mesenchymal stromal cell; AT-MSC, adipose tissue-derived mesenchymal stem cells; BM-MSC, bone marrow-derived mesenchymal stem cells.

### Overview of MSC

MSCs are multipotential progenitor cells with an intrinsic ability to differentiate into mesodermal cell lineages.^7,8^ They can be isolated from a variety of sources including bone marrow,^9^ umbilical cord,^10^ adipose tissue,^11^ dental pulp,^12^ and virtually from any vascularized tissue.^13^ When comparing the biological properties of MSCs from different sources, some studies have reported these to be similar^14-16^ whilst others have reported differences in protein expression profile, cytokine production, differentiation capacity,^17^ surface antigen expression, and immunomodulatory activity.^18,19^ These differences in biological properties result from heterogeneity in culture expanded MSC population which varies with MSC manufacturing variables including donor variation,^20^ isolation technique, culture protocol, media used, passage number as well as tissue origin the cells.^21^ This poses challenges when comparing the results of different studies with MSC and predicting the therapeutic potency of the MSC product for a specific clinical application. To set minimal standard criteria, the International Society of Cellular Therapy (ISCT) decreed 3 phenotypic criteria to define human MSC in 2005.^22^ These include (1) plastic adherence when maintained under standard culture conditions; (2) expression of CD105, CD73, CD90, and lack of expression of CD45, CD34, CD14, or CD11b, CD79 alpha, or CD19 and HLA-DR surface molecules; and (3) the ability to differentiate into osteoblasts, adipocytes, and chondroblasts. These criteria reflect the “stemness” of MSC and not their immunomodulatory and regenerative properties. Hence, ISCT have updated the MSC definition to include further 2 criteria (1) tissue origin and (2) associated functional assays informed by intended therapeutic mode of action.^23^

In addition to their progenitor properties, MSCs have an ability to modulate the adaptive and innate immune systems by suppressing T-cell activation and proliferation, suppressing dendritic cell maturation, reducing B-cell activation and proliferation, inhibiting proliferation and cytotoxicity of NK cells and promoting generation of regulatory T cells.^24,25^ MSCs also have the ability to switch macrophages between pro- and anti-inflammatory phenotypes which is in part mediated by phagocytosis.^26^ Dazzi et al^27^ demonstrated this effect in a graft versus host disease (GvHD) model, whereby apoptosis of infused MSCs by host macrophages was found to be central to the initiation of MSC-induced immunosuppression. Indole 2,3 dioxygenase (IDO) released by the apoptotic MSCs was reported to be the key soluble factor for immunosuppressive activity. More recently, De Witte et al^28^ demonstrated the rapid clearance of infused MSCs was mediated via phagocytosis by monocytes. In vitro experiments confirmed that human CD14^++^/CD16^−^ classical monocytes polarized toward a non-classical CD14^++^ CD16^+^ CD206^+^ phenotype after phagocytosis of MSC, and subsequently expressed programmed death ligand-1 and IL-10. MSC-primed monocytes also induced Foxp3+ regulatory T-cell formation in mixed lymphocyte reactions. This study demonstrated that phagocytosis of MSC induces phenotypical and functional changes in monocytes, which subsequently modulated cells of the adaptive immune system.

Initial studies reported MSCs as relatively immune privileged as they do not express class II major histocompatibility complex (MHC) and/or other co-stimulatory molecules at high levels which facilitates their allogeneic use.^29^ Several pre-clinical and clinical studies have however challenged the degree to which MSCs are immune-privileged, by demonstrating an immune response following allogenic MSC transplantation.^29^ MSCs exposed to IFN-γ have demonstrated both MHC Class I and Class II expression.^30^ MSC-derived extracellular vesicles including exosomes and micro vesicles have also been identified as central to their trophic effects^31,32^ which will be discussed later in this review.

### Efficacy of MSCs in Experimental Models of Liver Injury and Disease

**Figure 1.**
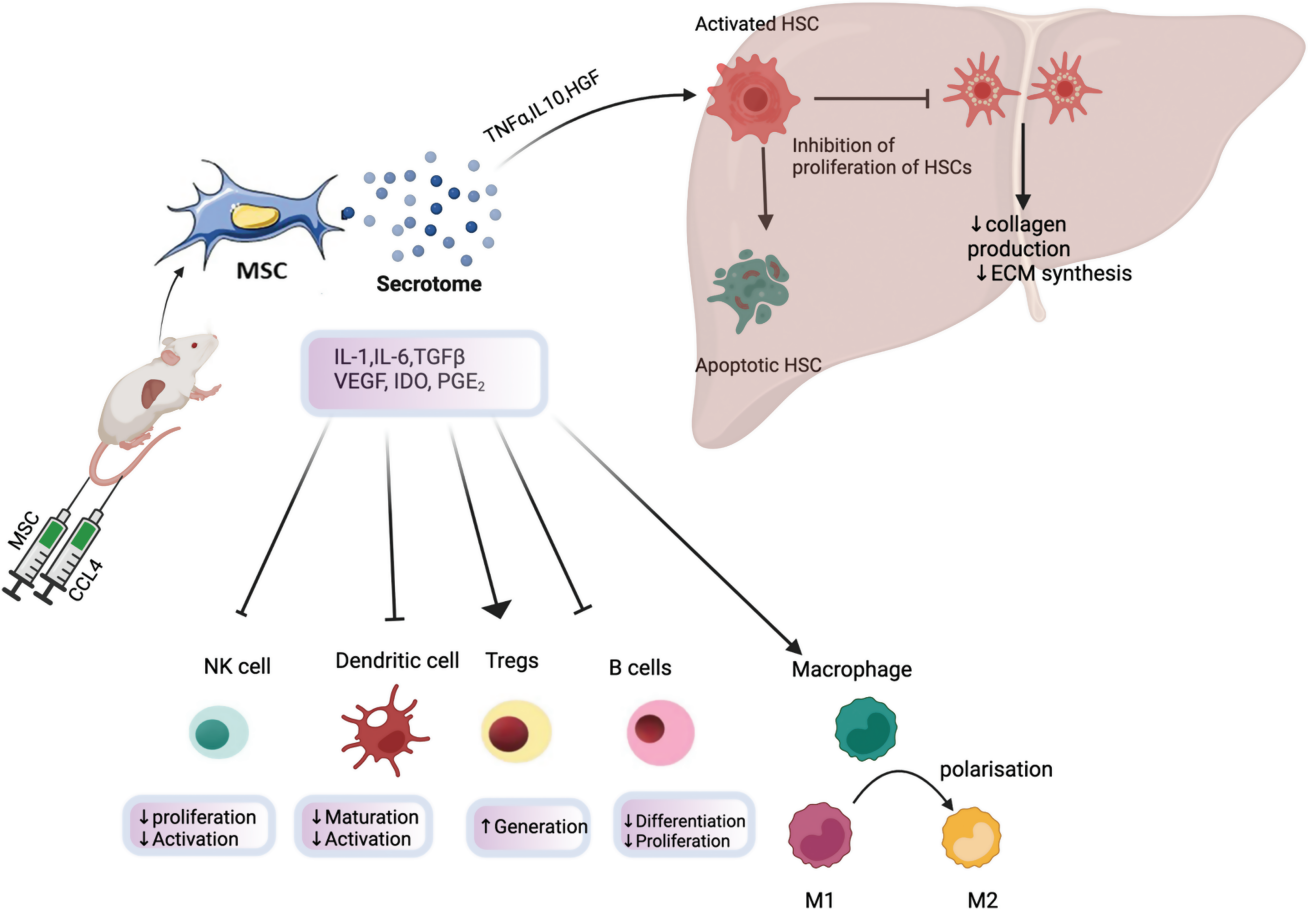
Effects of adoptively transferred MSCs in murine models of liver disease have been reported. MSCs release various soluble factors as part of its secretome including interleukin-1 (IL-1), interleukin-6 (IL-6), transforming growth factor β(TGF-β),vascular endothelial growth factor (VEGF), indoleamine 2,3 dioxygenase (IDO), hepatocyte growth factor (HGF),Tumour necrosis factorα (TNFα). These mediators modulate hepatic fibrogenesis through inhibition of proliferation of hepatic stellate cells (HSC) as well as through inducing apoptosis of HSCs. This results in decreased synthesis of collagen and reduced synthesis of extracellular matrix(ECM). MSC induced modulation of immune cells including natural killer cells (NK), regulatory T cells (T regs) etc reduces the hepatic infliltration of inflammatory cells and thus, exerts an anti-inflammatory effect . This immunomodulation also has an indirect antifibrotic effect. Polarisation of macrophages from proinflammatory (M1) to anti-inflammatory (M2) phenotype also plays a key role in this process.

Hepatic fibrosis represents the final common pathway of chronic inflammatory liver injury and is mediated by the activation of hepatic stellate cells (HSCs), the key effectors of fibrogenesis.^33,34^ The MSC secretome has been linked to therapeutic benefit by inhibiting liver fibrosis due to the production of transforming growth factor beta-isoform 3 (TNF-β3), tumor necrosis factor α (TNFα), hepatocyte growth factor (HGF),^35^ and IL-10, all of which inhibit the proliferation of ΗSCs (Fig. 1).^36,37^

The antifibrotic effects of adoptively transferred MSCs have been reported in various murine models of liver injury and fibrosis (Table 2). Qiao et al^38^ reported human bone marrow (hBM-MSC)-induced inhibition and apoptosis of activated HSCs in models of murine liver fibrosis. This was associated with increased expression of pro-apoptotic genes Bax and cleaved capase-3 protein Bax, which was proposed as part of the underlying therapeutic mechanism along with inhibition of NADPH oxidase pathway. The anti-inflammatory attributes of MSCs may also play an indirect role in exerting antifibrotic effects, with candidate soluble factors released by MSC including nitric oxide (NO), prostraglandin-E2 (PGE2),^39^ indoleamine 2,3 dioxygenase (IDO),^40^ interleukin-6 (IL-6), IL-10, and HLA-G. MSC can also modulate a range of other immune cell populations including their induction of regulatory T cells. Milosavljevic et al^41^ investigated the role of MSCs in modulating IL-17 signaling and its subsequent effect on CCL_4_ induced hepatic fibrosis in mice. Decreased serum levels of profibrogenic IL-17 were observed alongside increased levels of immunosuppressive and hepato-protective interleukin-10 (IL-10) and IDO following the administration of MSC. In a study by Fathy et al^42^ adipose tissue-derived MSC (ASC) along with eugenol preconditioned ASC were injected in CCL_4_ model of liver fibrosis. Eugenol is a natural compound with various concentration-dependent pharmacological activities including inhibition of NF-ΚB activation, promotion of cell cycle arrest, reduction in inflammatory cytokines, etc.^43,44^ Eugenol improved self-renewal, proliferation, and migration abilities of ASCs through upregulation of c-MET, Rex1, Oct4, and nano genes. Effective homing of E-ASCs also resulted in attenuation of expression of genes involved in inflammation including inducible nitric oxide (iNOS), monocyte chemoattractant protein-1 (MCP-1), cluster differentiation 163 (CD163), and tumor necrosis factorα (TNFα). Histological examination of the fibrotic livers demonstrated a marked reduction in inflammatory cell infiltrate, reduced hepatocyte damage, and a preservation of tissue architecture in the E-ASC group in comparison with ASC only or CCL_4_ only groups. Similarly, Chai et al^45^ reported an increase in anti-inflammatory cytokines including IL-4 and IL-10 alongside mobilization of liver resident macrophages (Kupffer cells) following administration of MSC in a murine model of liver fibrosis. Addition of IL-4 antibodies into coculture of MSCs and KCs resulted in decreased KC mobilization. MSC-induced polarization of proinflammatory macrophages (M1) to anti-inflammatory (M2) phenotype was proposed as a mechanism to reduce liver fibrosis.

**Table 2.** Efficacy of MSCs in murine models of liver disease.

Murine model	MSC source/route of infusion	Dose and time of treatment	Mediators	Effects	References
Fibrosis	Mouse AD-MSC alone or incubated with eugenol 2 × 10^6^ cells/well Tail vein	At week 5 4 groups (1) Control (2) CCL_4_ treated (3) CCL_4_+ MSCs (4) CCL4+ Eugenol preconditioned MSCs	↓ Fibrotic markers (Type III collagen, HA, hydroxyproline) ↓inflammatory cytokines	Effective homing of eugenol treated MSCs with attenuation of liver inflammation and fibrosis	Fathy et al. ^42^
	Human BM-MSCs Liver portal vein	8 x 10^6^ of hBM-MSCs after 11 weeks of CCL_4_	Apoptosis of activated HSCs mediated through NADPH oxidase pathway	↓ Liver fibrosis	Qiao et al. ^38^
	Mouse BM-MSCs and MSC-CM Tail vein	1 × 10^6^ after 24 hours of CCL4 and on 7^th^,14^th^ and 21^st^ day of experiment	↓ Serum IL-17 ↓ Th17 ↑ IDO, IL-10, CD4^+^FoxP3^+^	↓ Liver fibrosis and ↓ Liver inflammation	Milosavljevic et al ^41^
	Human UC-MSCs Tail vein	5 × 106 7 days after DMN treatment	↑IL-4 and IL-10 KC mobilization	↓Liver fibrosis	Chai et al ^45^
	Mouse BM-MSC Tail vein	2 doses 0.5 × 10^6^ Day 60 and 61	Cytokine induced ↑expression of IDO and iNOS ↓ MMP-2	MSC induced ↓ fibrosis MSc effects negated in mice pre-treated with dexamethasone.	Chen et al ^56^
FHF/ ALF	Mouse BM MSC Tail vein	In FHF: After 5 h of TAA. 200 μL MSC-CM. or 1 × 10^6^ MSC In CCL_4_: 1 × 106 MSC or 200 μL twice/week for 3 weeks at 6 weeks of CCL4 infusion	↑ Tregs ↓Th1, Th17 Down regulation of HSC and infiltrating macrophages	Inhibition of hepato-cellular apoptosis Stimulate liver regeneration	Huang et al 2016 ^57^
Cirrhosis	Human MSC	24 h after induction of FHF 2 × 10^6^ hMSCs or MSC-CM	↓Attenuation of leukocyte migration to liver	↓ Liver injury. Pan-lobular leukocyte invasion and hepatocellular death	Parekkadan et al .^58^
	Mouse BM-MSCs and BMMs Tail vein	At 8 weeks: injection of 1 × 10^6^ MSCs or id-BMMs; or 5 × 10^5^ MSCs and id-BMMs	↑ mRNA of MMP-13 SDF-1,IL-10 and IL-13, PGE2	Reduced liver fibrosis. Stronger effect in MSC-id MMs then MSC alone. Improved liver function.	Watanabe et al ^59^
	Mouse ADSCs Splenic vein injection	8-week mice injected with 1 × 10^5^ ADSCs	↓ Ratio of CD8^+^/CD4^+^ cells ↓Intrahepatic infiltration of CD11^+^ and Gr-1^+^ cells	↓Liver fibrosis	Seki et al ^11^
PBC	Mouse BM-MSC	1 × 10^6^ cells at week 16	IFNγ↓ CD4^+^FoxP3^+^, TGFβ↑	↓Liver injury, inflammation, inflammatory cell infiltration	Wang et al ^60^
Acute liver injury/ hepatitis	Mouse and Human MSCs and MSC CM Tail vein		↓ TNFα, IFNγ, IL-4 iNOS and IDO dependant attenuation of cytokines	↓Liver injury	Gazdic et al ^61^
	Mouse AD-MSCs Tail vein	1 × 10^5^ ADSCs immediately after ConA injection	↓CD11b^+^ Gr-1^+^, F4/80^+^ cells	↓ALT and LDH ↓Hepatocyte necrosis	Higashimoto et al ^62^
NASH	Mouse BM-MSC i.v. tail	1 × 10^6^ cells at weeks 6 and 7	↓CD4^+^IFNγ,IL-6^+^	↓Steatosis, ballooning lobular inflammation and hepatic fibrogenesis	Wang et al ^63^

Abbreviations: ADMSCs, adipose tissue MSCs, NADPH, nicotinamide adenine dinucleotide phosphatase; antifibrotic factor, DMN, MMP-13; SDF-1, chemoattractant factor; IL-10 () IL-13, anti-inflammatory; PGE2, prostaglandin E2.

MSCs have also been reported to inhibit epithelial to mesenchymal transition (EMT) and exert an antifibrotic effect in liver fibrosis models. EMT refers to a mechanism where by certain resident liver epithelial cells undergo biological changes to assume a mesenchymal phenotype and contribute to the fibrogenic process in the injured liver.^46,47^ Some of these epithelial-derived mesenchymal cells, however, may subsequently undergo mesenchymal to epithelial transition and ultimately become hepatocyte and cholangiocytes. Balance between EMT/MET has therefore been suggested to determine the outcome of liver injury.^47^ There is an inherent difficulty in establishing EMT as a mechanism of liver fibrosis and its significance in the outcomes of liver injury.^48^ Furthermore, existing data available from murine studies that can help understand the role of EMT in liver fibrosis/repair is confounded by different models of injury and examination of different outcomes at different time points across the studies.^47^ Nonetheless, antifibrotic effect of human UC-MSC derived exosomes through inhibition of EMT of hepatocytes has been demonstrated in CCL_4_ model of liver fibrosis.^49^ Inactivation of (TGFβ)-1/Smad signaling pathway was shown to be involved. In an another study, MSC-induced amelioration of surgery-induced liver damage and inhibition of EMT has been reported in a pig liver resection model.^50^ Mechanistic analysis showed modulation of thrombospondin-1/TGF-β to play a key role. Thrombospondin-1 secreted from thrombocytes and non-parenchymal cells is linked with TGF-β production and EMT. In the pig resection model, it was proposed that MSC-derived soluble factors inhibited thrombospondin-1 secretion with resultant reduction in active TGF-β and subsequent attenuation of liver damage.^50^

While data from these studies along with other studies listed in Table 2 support indirect antifibrotic effects of MSCs, there are other studies that report no effect of MSC on liver fibrosis when administered at, or after, cessation of liver injury.

Carvalho et al^51^ injected BM-MSC at a dose of 1 × 10^7^ cells in a model of severe chronic liver injury (15 weeks of CCL_4_ and 14 weeks of alcohol diet) and demonstrated no improvement in liver injury biomarkers with no difference in histological results between MSC treated and placebo group. Mannheimer et al^52^ reported similar findings with MSC isolated from cirrhotic rats. Hepatic injury was induced in a bimodal pattern with alcohol infused diet and injection of CCL_4_ for a prolonged period of time (14 weeks) but no difference in fibrosis was noted in the treated group in comparison to controls. This highlights the impact of source of MSC and duration of liver injury on therapeutic potential of MSC. Similarly, Briquet et al^53^ also reported no antifibrotic effects of MSC when administered after cessation of liver injury. They injected hBM-MSC, UC-MSC, and liver MSC in NOD/SCID/IL-2Rg null (NSG) mice after 4 weeks of CCL_4_-induced fibrosis. They reported no therapeutic effect on liver fibrosis, but this study published in 2014 was later retracted based on misuse and misrepresentation of a peer’s scientific data.

Profibrotic effects of MSC-like cells have also been reported. Genetic lineage tracing of tissue resident perivascular GLi^i+^ cells shows them to be MSC-like cells which contribute significantly to myofibroblast generation in CCL_4_-nduced liver fibrosis.^54^ Baertschiger et al^55^ reported human BM-MSC introduced via intrahepatic injection in mice undergoing liver regeneration or fibrosis contribute to myofibroblast formation.

In summary, it can be concluded that the direct anti-fibrotic actions of MSCs remain controversial, with the more likely case being that any such effect is mediated indirectly through an effect on liver inflammation.

### Autologous Versus Allogenic MSC Transplantation

Both autologous and allogeneic MSCs have been studied in preclinical and clinical studies, and despite the relative ease in obtaining autologous MSCs, this often comes at a high cost of preparation for a single recipient. There is also a time-critical aspect of obtaining and expanding enough cells for transfusion, raising logistical challenges for their use in treating acute diseases. It is also difficult to obtain sufficient autologous MSCs from some patients, for example, ASC from thinner patients. MSCs from elderly donors have been shown to lack in proliferation and differentiation, thus potentially possessing less regenerative properties.^64^ In contrast, allogeneic MSCs are usually obtained from young healthy donors, are readily available, and can be cryopreserved, stored, and quickly thawed prior to administration. This process also allows for quality assurance of MSCs and reduces their overall cost. Overall, cryopreserved allogeneic MSCs offer many advantaged compared to autologous MSCs as regards time, cost production, and quality assurance.

## Emerging Role of Extracellular Vesicles

Bruno et al^65^ fractioned human MSC conditioned media (MSC-CM) by ultracentrifugation and demonstrated that MSC-like effects were retained within the cell-free supernatant and associated with 80 nm to 1 μm spherical moieties. These were described as microvesicles and were able to suppress murine acute renal tubular injury in vivo.^65^ The microvesicle fraction also suppressed apoptosis and enhanced tubular epithelial cell proliferation in vitro to a similar extent as MSC. Lai et al^66^ used high performance liquid chromatography (HPLC) for size exclusion and enriched a fraction comprised of particles with a hydrodynamic radius of 55-66 nm. These particles were named exosomes based on the presence of exosome associated proteins such as CD9, CD81, and Alix and resulted in a reduction in size of an infarct in a murine model of acute myocardial infarction (AMI). Similar effects have been observed in a previous study with MSC and MSC conditioned media MSC-CM^67^ and since then there is growing preclinical evidence to support the role of MSC-derived EVs as therapeutic agents.^68^

### Characteristics of MSC-EV

**Figure 2.**
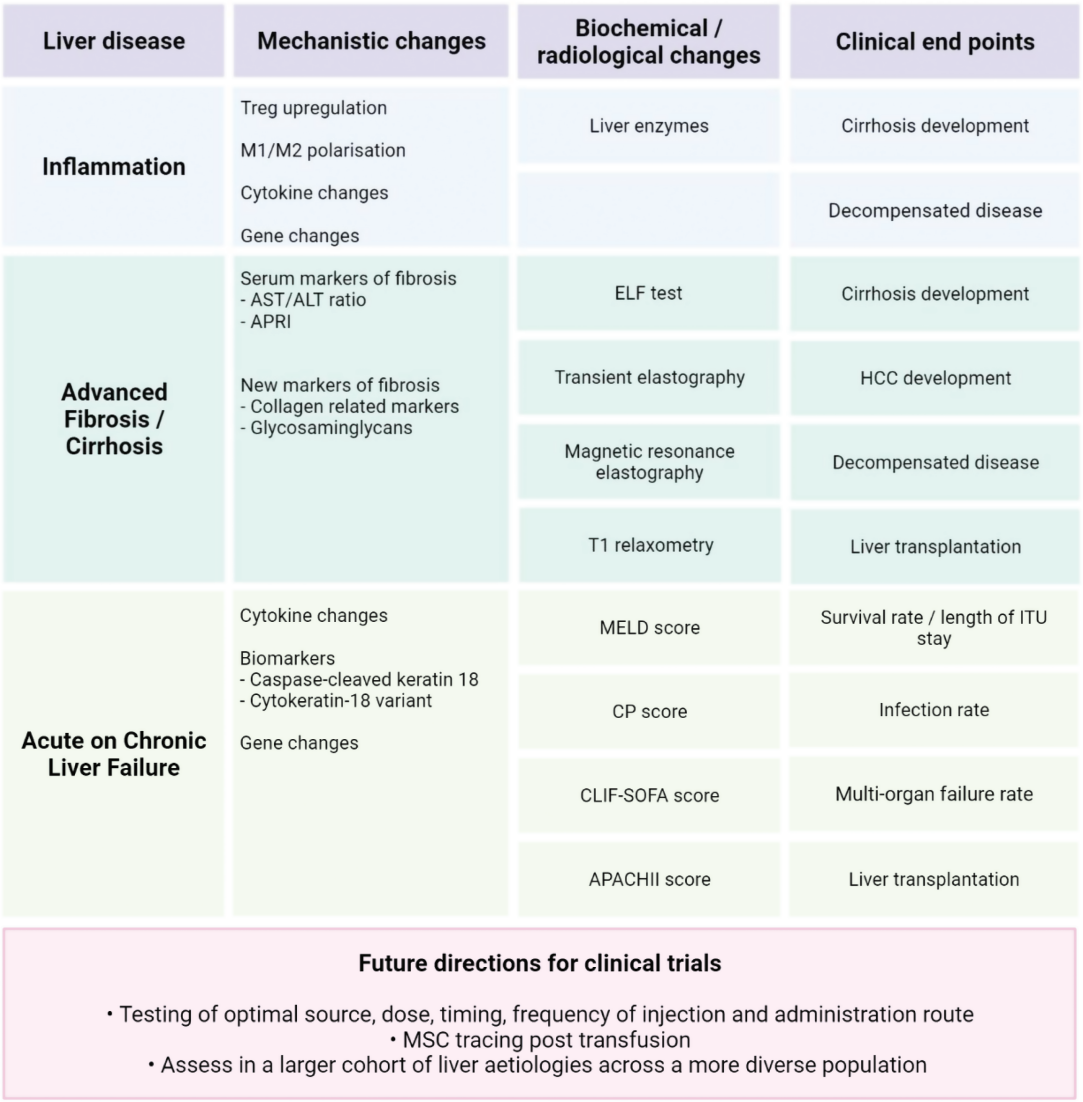
MSC and clinical trials. ALT, alanine aminotransferase; APACHII, acute physiology, and chronic health evaluation; APRI, AST to platelet ratio index; AST, aspartate aminotransferase; CLIF-SOFA, chronic liver failure-sequential organ failure assessment; CP, Child-Pugh score; ELF, enhanced liver fibrosis test; HCC, hepatocellular carcinoma; ITU, intensive therapy unit; M1, macrophage 1; M2, macrophage 2; MELD, model for end-stage liver disease; MSC, mesenchymal stromal cell; T1, longitudinal relaxation time; T reg, regulatory T cells.

MSC secrete different classes of EV including exosomes,^69^ microparticles,^70^ and microsomes,^65^ and each subset is defined by its physical, biochemical, and biogenetic characteristics.^69,71^ At present, exosomes are probably the best described EV particles with specific surface markers^72^ and contents that include mRNA, miRNA, and assorted proteins which in turn can modulate function of target cells.^32,73^ EV exert their effects through cell signaling,^74^ alterations in cell metabolism,^75^ and via intercellular communication.^76^ Pre-treatment of MSC-EV can also impact their content and subsequent therapeutic action which requires further research (Fig. 2).^77^

A wide array of therapeutic effects has been attributed to such mRNAs in experimental models, for example, the transfer of insulin like growth factor-1 receptor (IGF-1R) mRNA from exosomes to cisplatin-damaged proximal tubular epithelial cells sensitizes them to the reno-protective effects of locally produced IGF-1.^78^ In other studies, mRNA from MSC exosomes has been shown to play a role in inhibiting tumor growth,^79,80^ reducing cardiac fibrosis,^81^ stimulation of axonal growth from neurons,^82^ and angiogenesis.^83^ An in vivo study demonstrated that miRNA-223 from MSC-derived exosomes was critical in delivering MSC-induced cardio protection in sepsis,^84^ which was achieved through downregulation of the inflammation related genes Sema3A and stat3.^85^

The contents of MSC EV and their subsequent therapeutic effects can be a function of their origin; for example, bone marrow-derived MSC EV contain cystinosin (CTNS), a cystine efflux channel in the lysosomal membrane, which can reduce cystine levels when cocultured with renal tubular cells from patients with cystinosis.^86^ Similarly, adipose tissue-derived MSC (ADSC) contain up to four-fold higher concentration of neprilysin, an enzyme that degrades β-amyloid (Aβ) peptide in brain and which is associated with Alzheimer’s disease, than BM-MSCs. Transfer of ADSC-derived exosomes into N2a cells (neuroblastoma cell line) decreased intracellular Aβ levels.^87^ Due to the lack of an agreed methodology for EV preparation, the international society for extracellular vesicles (ISEV) released a position statement to define minimal criteria recommend for characterization of purified EVs^88^ which included semi-quantitative analysis of the EV protein composition for typical EV marker proteins such as CD9, CD63, CD81, Alix, and TSG101, size analysis and analysis of their morphology. ISEV also agreed on naming all experimentally derived vesicles as EV.

### Role of MSC-EVs in Liver Injury

Recent research has shown that MSCs produce a significant amount of EVs, which has been proposed to be one of the mechanisms by which MSCs exert their various therapeutic effects in liver injury.^89^ In light of this, Newman et al^90^ proposed that MSCs can be used to take advantage of the critical role of EVs in liver cell communication by transporting a variety of macromolecules, such as connective tissue growth factor (CTGF), which facilitates the interaction between parenchymal cells and non-parenchymal cells such as hepatocytes, hepatic stellate cells (HSCs), and Kupffer cells.

Fiore et al^91^ demonstrated that the delivery of hepatocyte-derived EVs enhanced hepatocyte proliferation, suppressed cell death, and accelerated liver regeneration in rats following 70% hepatectomy. This was hypothesized to be caused by EVs delivering RNA to target cells through fusion with target hepatocytes, which then transferred the cargo.^92^ For example, hepatocyte-derived EVs provide neutral ceramidase and sphingosine kinase 2 (SK2) to hepatocytes, which induces sphingosine-1-phosphate (S1P) respectively.^93^ These findings suggest that MSC-derived EVs may have the potential to be a new therapeutic for both acute and chronic liver damage.^89^

MSC-EVs express CD40L, which activates macrophages and changes their phenotype from anti-inflammatory to pro-inflammatory,^92^ which may play an important role in liver regeneration and resolution of liver fibrosis. When exposed to pro-inflammatory cytokines, regional growth factors, and microbial metabolites, miR-27a, which is abundant in naïve monocytes, is known to cause M2 polarization and hence a switch to an anti-inflammatory micro-environment.^91^

Additionally, during liver injury, hepatic stellate cells (HSCs) and EVs produced by MSC interact resulting in the release of connective tissue growth factor (CCN2),^90^ which by binding integrin v3 or v5 with heparan sulfate proteoglycan ligands, can signal to endothelial cells to modulate activation.^91^ On the other hand, MSC-EVs affect target cells via activating the transcription factors Twist-1, miR-214, and miR-199a-5p.^89^ Despite numerous preclinical studies on liver fibrosis with MSC-EVs, it remains unclear whether MSCs-EV stimulate local damage liver cells.

### MSC-EVs in Liver Disease Models

MSC-EVs were initially investigated as a potential therapy for accelerating liver regeneration in the model of 70% hepatectomized rats.^94^ Recent murine studies (listed in Table 3) have demonstrated the paracrine effects of MSC EV, with Haga et al^95^ demonstrating reduced hepatic injury, reduced apoptosis, and modulated cytokine expression after systemic administration of BM derived MSC-EV in models of fulminant hepatic failure. Chen et al^96^ reported similar anti apoptotic ability of MSC EV with improved liver function, when human menstrual blood stem cell-derived exosomes (MenSC-Ex) were injected into the tail vein of mice 24 h before D-GalN/LPS induced fulminant hepatic failure (FHF). Hepatic regeneration and anti-apoptotic effect has also been reported in a murine model of drug induced liver injury by Tan et al,^97^ with upregulation of Bcl-xL protein expression implicated as the underlying mechanism. Glutathione peroxidase1 (GPX1) derived from human umbilical cord MSC (hucMSC) exosomes was reported to elicit antioxidant and anti-apoptotic effects in CCL4 induced liver failure when administered as a single dose via tail vein. Similarly, Li et al^89^ demonstrated amelioration of CCl_4_ induced hepatic inflammation and liver fibrosis in mice when treated with hucMSC-EV. The underlying mechanism involved inactivation of TGF-β1/Smad signaling pathway, reduced expression of collagen I and III, and inhibition of epithelial to mesenchymal transition (EMT). In another experiment by Zhang et al.^98^ MSC-EV pre-treated with TNFα (T-exo) were administered after an hour of lipopolysacharride(LPS)/D-galactosamine (D-GalN) induced liver injury. T-exo were reported to reduce ALT, AST, and pro-inflammatory cytokine levels and inhibited the activation of NLRP3 inflammation associated pathway proteins and thus, promoted tissue repair.

**Table 3.** Effects of MSC exosomes in murine studies.

Source of exosome	Liver disease model	Effects	Mediators/pathway	Ref.
MenSC-Ex	Fulminant hepatic failure (FHF)	Anti-apoptotic Improved liver function ↓ Necrosis and inflammation	↓ TNFα, IL-6 and IL-1β.	Chen et al ^96^
Murine or human BM-MSC Exosomes	FHF	Anti-apoptotic/reduced hepatic injury Modulation of cytokine expression	y-RNA-1	Haga et al ^95^
hiPSC-MSC-Exo	Hepatic ischemia-reperfusion injury	Anti-apoptotic Anti-oxidative stress response	↓ Hepatocyte injury markers in treatment group ↓TNFα, IL-6, HMGB1. ↓In apoptosis protein (Bax, Caspase-3) and GSH, GSH-Px, and SOD higher than in control group	Nong et al ^99^
Human foetal tissue derived MSC-exo	CCL4-induced hepatic injury followed by xenobiotic-induced liver injury	↓Hepatic injury ↑Hepatocyte proliferation and inhibition of apoptosis	↑ Cell viability in treatment group. ↑ In proliferating protein pANCA and cyclin D1 Upregulation of Bcl-xL protein	Tan et al ^97^
huc-MSc-Exo	CCL4-induced hepatic failure	Antioxidant and anti-apoptotic effect	Induction of ERK ½ phosphorylation and inhibition of IIKB/NFkB/casp9/3 pathway Delivery of GPX-1 protein	Yan et al ^100^
Huc-MSCs-Exo	CCL4-induced liver fibrosis	↓Hepatic inflammation ↓Liver fibrosis	↓Collagen I and III ↓TGF-β1 Inactivation of TGFβ/Smad2 pathway Inhibition of EMT	Li et al ^49^
T-Exo and hucMSC-Ex	LPS+D-GalN ALF	↓ ALT, AST, proinflammatory cytokines	Inhibit NLRP3 proteins	Zang et al ^98^
ADMSCs EXO	HCC	HCC suppression	↑ Nk-T cell ↑ ADC ratio	Ko et al ^101^

Abbreviations: MenSC-Ex, menstrual blood-derived exosomes; TNFα, tumor necrosis factor; BM-MSC, bone marrow MSC; hiPSC-MSC exo, human-induced pluripotent stem cell-derived MSCs Exo; HMGB-1, human mobility group box-1; GSH, glutathione; GSH-PX, glutathione peroxidase; SOD, superoxide dismutase; pANCA, proliferating cell nuclear antigen; huc-MSC exo, human umbilical cord MSC exosome; GPX-1, glutathione peroxidase 1; ERK, extracellular signal regulated kinase^64^; Bcl, B-cell lymphoma; EMT, epithelial-to-mesenchymal transition; CP-MSCs, chorionic plate MSCs; 9LPS, lipopolysaccharide; D-GalN, D-galactosamine; ADMSCs, adipose-derived MSCs; NK-T cells, natural killer T cells; ADC, apparent diffusion coefficient; HCC, hepatocellular carcinoma.

Data from these studies and those summarized in Table 3 suggest MSC-EVs may be effective in inhibiting hepatocyte apoptosis, support hepatocyte function, promote hepatocyte proliferation and in addition, modulate inflammatory response by preventing immune cell infiltration and/or stimulation of inflammatory cytokines. While these murine studies make a case for MSC EVs as an alternative to cell-based therapies for liver disease, further investigations to elucidate the optimal route and dose of administration, and precise mechanism of biological actions are required. It should be noted that majority of these studies were of short duration of liver injury 4-6 weeks with immediate administration of MSC EV.

### Limitations of Experimental Models

Tsiapalis et al^102^ claimed that finding reliable, reproducible, and robust approaches for isolating and purifying therapeutic EVs and their mass manufacture at the cGMP quality for clinical application poses a significant challenge. Additionally, little is understood about EVs’ biogenesis, uptake, and cellular activity, making it unclear how they may affect therapeutic outcomes, with Maji et al,^103^ indicating that EV contents can vary with cell type and environmental influences. It is necessary to conduct *in vivo* assessments of their pharmacokinetics, biodistribution, dose-escalation, toxicity, and immunogenicity, in addition to providing evidence of the effectiveness and potency of any EV-based therapeutics. Additionally, identifying which EV subpopulations among the heterogeneous populations will be important and indeed the classification into several types is still under investigation. Additional studies are also necessary to determine the most effective therapeutic dosages and delivery methods for clinical use in the future to ensure improved EV homing and targeting of injured liver.^104^ The use of MSC-EVs in clinical settings will require resolution of several issues, including large-scale production and isolation methods, (ii) methods for rapid and accurate quantification and characterization of EV, (iii) precise content characterization of the cargo, (iv) pharmacokinetics, targeting and transfer mechanisms of EV to the target sites, and (v) safety profiling.

## Hepatic Pathogenesis and Role of MSCs

Chronic liver injury leads to fibrosis, cirrhosis and hepatocellular carcinoma development and is caused by a range of factors including hepatitis B or C, cholestasis (primary biliary cirrhosis, primary sclerosing cholangitis), alcohol related liver disease and non-alcoholic steatohepatitis. Liver fibrosis occurs as a result of acute and/or chronic cellular injury mediated via activation of hepatic stellate cells to myofibroblasts. HSCs are the primary effector cells that lead to deposition of collagen in extra-cellular matrix (ECM), and which also interact with other immune cells, secreting cytokines and chemokines. Other mechanisms of fibrosis have also been described with ECM mechanical stiffness also activating further HSCs.^105^

The role of dendritic cells is less well understood; however, they can activate NK cells which in turn mediate HSC apoptosis causing downregulation of inhibitory MHC class 1 molecules. Other mechanisms include adipokines as key mediators in fibrogenesis particularly in the setting of NAFLD, where Leptin has been shown to promote HSC fibrogenesis and enhance TIMP-1 expression which is associated with increased leptin signaling. It also partially suppresses peroxisome proliferator-activated receptor-γ (PPARγ) anti-fibrogenic nuclear receptor that can reverse HSC activation and maintain HSC quiescence.^106^

### MSC Mechanism of Action in Clinical Settings

Several mechanisms of actions of MSCs have been described with Kampera et al^107^ summarizing the 3 main mechanisms of action as: 1. Living cell expansion and differentiation into mesodermal tissues 2. Close interactions with neighboring cells via cell-to-cell contact and release of paracrine factors and extracellular vesicles 3. Apoptotic phenomena involving MSCs and immune cells, ie, efferocytosis of cellular debris, leading to functional polarization of phagocytic cells toward inhibitory phenotypes.

Several studies in vivo have suggested that human MSCs can differentiate into hepatocyte-like cells when transplanted.^108-115^ Seo et al^116^ retrieved human adipose-derived MSCs in the liver of CCl-4-injured severe combined immunodeficiency mice after intravenous injection and differentiated to hepatocyte-like cells. Chamberlain et al^117^ injected human-derived MSCs into pre-immune fetal sheep in the absence of liver injury—this resulted in the generation of hepatocytes 70 days after xenotransplantation. These studies suggest that MSC hepatocyte-like derived cells may replace damaged hepatocytes in liver disease. Importantly, the *in vivo* signals orchestrating trans differentiation are not completely understood at present. The overall contribution of this mechanism to ameliorating liver injury and repair is likely low though.

The immunosuppressive role of MSCs has been confirmed both in pre-clinical and clinical settings, where persistent inflammation drives liver injury through infiltration with T cell, B cell and monocytes. A reduction in inflammation will reduce injury and also facilitate liver regeneration, as potentially achieved by MSCs suppression of T lymphocyte activation, proliferation and cytotoxicity.^118^ Other mediators of MSC suppressive effects include secretion of prostaglandin E2 that promotes IL-10 secretion by dendritic cells, increase in regulatory T cells and decrease in TNF-α, INF-γ, and IL-4; indoleamine 2,3-dioxygenase with INF-γ stimulation, leading to inhibition of T-cell proliferation.^35^ Selmani et al^119^ also demonstrated that HLA-G5 human leukocyte antigen protein was produced by MSCs, leading to a reduction in T-cell proliferation and an increase in T-cell numbers.

Chemokines and cytokines secreted by MSCs during both acute and chronic liver injury may also play a significant role in reducing inflammation and hepatocytes apoptosis. Poll et al^120^ demonstrated that administration of MSC medium downregulated IL-1β, TNF-α and IL-6. It increased levels of IL-10. Overall, this resulted in lower lymphocyte infiltration in the liver, reduced hepatocyte apoptosis and increased hepatocyte proliferation.

The main anti-inflammatory signals of MSCs in liver disease that have been described in the literature include secretion of epidermal growth factor,^121^ TNF-α secretion inhibiting stellate cell proliferation and collagen type 1 synthesis,^36^ promotion of hepatic stellate apoptosis through nerve growth factor secretion.^122^ Higashyimama et al^123^ also demonstrated extracellular matrix degradation via matrix metalloproteinase-9 expression. The pro-inflammatory cytokines that have been described are TGF-β 1 and 3, IL-6, monocyte chemoattractant protein-1, macrophage inflammatory protein-1 α and β.^120,124,125^

Of note the majority of MSC transplanted cells are entrapped in the pulmonary circulation and subsequently cleared from there^126^ which reduces their homing capability and engraftment within target tissues. Paracrine and apoptotic phenomena are therefore more likely to play a significant role; however, the exact mechanisms remain unknown.

Although various mechanisms for MSCs mitigating hepatic fibrosis directly have been described, the case is not fully proven, thus the MSC mechanism of mitigating fibrosis directly remains uncertain.

### Therapeutic Mechanisms of MSC in the Injured Liver

The replacement of injured tissue by differentiating into different cell lineages and the modulation of immune responses are 2 fundamental mechanisms contributing to MSCs’ therapeutic potential,^127^ although the latter is by far the most important. According to Fan et al,^128^ studies conducted *in vitro* and *in vivo* have demonstrated that MSCs can modulate innate and adaptive immune systems by reducing T-cell activation and promoting T-cell development toward a regulatory phenotype. Indeed, Eom et al^129^ stated that MSCs release soluble proteins such as transforming growth factor-1 (TGF-1), indoleamine-pyrrole 2,3-dioxygenase (IDO), nitric oxide (NO), interleukin-10 (IL-10), a potent anti-inflammatory cytokine, prostaglandin E2 (PGE2), as well as extracellular vesicles (EVs), which have immunomodulatory functions and are therapeutic in many disease models.^130^

Furthermore, several studies indicate that MSCs may be important in macrophage polarization, encouraging differentiation toward the M2 phenotype both *in vitro* and *in vivo*. At the same time, MSCs can reduce NK cell proliferation and cytotoxicity.^131^ In addition, Protein kinase B, extracellular receptor kinase (ERK), and the mitogen-activated protein kinase (MAPK) axis all seem to be elements of various molecular mechanisms that drive MSC-EV-based therapy. Likewise, the Wnt/-catenin signaling pathway, which is important for tissue repair and cell destiny, might also be involved in EV-mediated tissue regeneration.^132^

Several studies have shown that MSCs can travel across vascular endothelial cells to injured tissue locations and engraft there. Eom et al^129^ reported that MSC homing could be divided into 2 main categories: non-systemic, which refers to the local transplantation of MSCs at the injured site, and systemic, which corresponds to the release of homing-promoting molecules from injured tissue.^132^ Adhesion molecules that are expressed on the MSC surface help in adhesion. Rolling, activation, firm attachment, crawling, and transendothelial migration are the 5 sequential processes that constitute MSC homing.^129^ According to numerous studies, hyaluronic acid (HA) may interact as a binding site for the CD44 receptor on activated endothelial cells with P-selectin on the MSC surface, promoting MSC homing and demonstrating rolling motion. At the same time, chemokines such as stromal cell-derived factor-1 (SDF-1) that are produced after damage can interact with C-X-C-motif chemokine receptors such as (CXCR4) and (CXCR7) on MSCs to activate integrin adhesiveness, which in turn can facilitate MSC migration^19^ MSC transportation and homing are significantly influenced by the shift to high affinity, which occurs once the chemokine attaches to its receptor and the cell membrane and binds to the integrin tail. A critical component of the firm adhesion process is the tight adherence between MSCs and vascular endothelial cells, which is promoted by the expression of VCAM1 intercellular adhesion molecule 1 (ICAM1) by activated MSCs.

After establishing firm endothelial adhesion, MSCs can help generate actin filaments and pseudopodia and subsequently promote cytoskeleton remodeling by forming intracellular linker molecules by activating the CCR2 signaling pathway.^93^ Because of this, MSC crawl along the inner wall of blood vessels whilst being subjected to a chemo-tactic gradient to find the best site for trans-endothelial migration.^133^ By secreting MMPs to complete trans-endothelial migration, this mechanism enables MSCs to damage the endothelial basement membrane. Of note, urokinase-type plasminogen activator (uPA), which breaks down ECM components, has been discovered in prominent pseudopodia of MSCs.

## Clinical Applications in Liver Disease

The use of MSC in liver disease has been the focus of many clinical trials, as summarized in Table 4, with applications mainly targeting patients with advanced fibrosis/cirrhosis rather than those with active inflammation. This merits scrutiny as MSC are focused on their anti-inflammatory actions which are more relevant in settings of MSC’s major mechanisms of action are focused on their anti-inflammatory actions, which are more relevant in settings of their anti-inflammatory actions which are more relevant in inflammatory rather than fibrotic disease.

**Table 4.** Clinical trials using MSCs to treat liver disease.

Study	Year	Liver disease	Type of clinical trial	Duration of follow-up (month)	Patients, sample size (MSC/cohort)	Source of MSC	Injection route	Dose	Primary outcomes
Mohamadnejad et al^154^	2007	Decompensated liver cirrhosis	Case series	12	4/0	Autologous BM	Peripheral vein	31.7 × 10^6^	MELD score and creatinine
Kharaziha et al^155^	2009	Liver cirrhosis	Cohort	6	8/0	Autologous BM	Portal vein (*n* = 6) Peripheral vein (*n* = 2)	3.0-5.0 × 10^7^	MELD score, prothrombin, and creatinine
El-Ansary et al^156^	2010	Decompensated liver cirrhosis due to HCV or HBV	Case-control	6	12/6	Autologous BM	Intra-splenic (*n* = 6) Peripheral vein (*n* = 6)	1.0 × 10^6^	Creatinine, prothrombin time, albumin, bilirubin, and MELD score
Amer et al^136^	2011	Decompensated liver cirrhosis due to HCV	Case-control	6	20/20	Autologous BM	Intra-splenic (*n* = 10) Intra-hepatic (*n* = 10)	2.0 × 10^7^	MELD score, ascites, peripheral oedema, Child-Pugh score, albumin, and fatigue
Peng et al^146^	2011	ACLF caused by HBV	Case-control	12	53/05	Autologous BM	Hepatic artery	1.0 × 10^6^	MELD score, prothrombin time, albumin, and bilirubin
El-Ansary et al^137^	2012	Decompensated liver cirrhosis due to HCV	Case-control	12	15/10	Autologous BM	Peripheral vein	1.0 × 10^6^	MELD score and albumin
Shi et al^147^	2012	ACLF associated HBV	Case-control	12	24/19	Allogeneic UC	Peripheral vein	0.5 × 10^6^	MELD score, albumin, prothrombin time, bilirubin, ALT, and survival rates
Zhang et al^138^	2012	Decompensated liver cirrhosis due to HBV	Case-control	12	30/15	Allogeneic UC	Peripheral vein	0.5 × 10^6^	MELD score, albumin, bilirubin and ascites
Amin et al^157^	2013	Chronic HCV – associated liver cirrhosis	Cohort	6	20/0	Autologous BM	Intra-splenic	1.0 × 10^7^	MELD score, albumin, prothrombin time, bilirubin, AST, and ALT
Mohamadnejad et al^134^	2013	Decompensated liver cirrhosis	RCT	12	15/12	Autologous BM	Peripheral vein	1.0 × 10^6^	No improvement
Wang et al^145^	2013	UDCA-resistant PBC	Cohort	12	7/0	Allogeneic UC	Peripheral vein	0.5 × 10^6^	Alkaline phosphatase and GGT levels
Jang et al^158^	2014	Alcohol-related liver cirrhosis	Cohort	6	11/0	Autologous BM	Hepatic artery	5.0 × 10^7^	MELD score and liver histology
Salama et al^140^	2014	Chronic HCV—associated liver cirrhosis	RCT	6	20/20	Autologous BM	Peripheral vein	1.0 × 10^6^	MELD score and Child-Pugh score
Wang et al^144^	2014	UDCA-resistant PBC	Cohort	12	10/0	Allogeneic	Peripheral vein	3.0-5.0 × 10^5^	ALT, AST, GGT and IgM
Xu et al^141^	2014	Chronic HBV-associated liver cirrhosis	RCT	6	27/29	Autologous BM	Hepatic artery	8.45 × 10^5^	MELD score improvement and reduction in IL-6, IL-17, and TNF-α levels
Kantarcioglu et al^159^	2015	Liver cirrhosis	No control group	6	12/0	Autologous BM	Peripheral vein	1.0 × 10^6^/kg	Partial improvement MELD score, no change in liver regeneration or fibrosis after 6 months
Suk et al^143^	2016	Alcohol-related liver cirrhosis	RCT	12	37/18	Autologous BM	Hepatic artery	5.0 × 10^7^	Histologic fibrosis and Child-Pugh score
Shi et al^151^	2017	Acute liver allograft rejection	RCT	5.5	14/13	Allogeneic	Peripheral vein	1.0 × 10^6^/kg	Liver function and graft histology, increased Treg/Th17 ratio, CD4 T-cell activation, elevated levels of TGF-b1 and PGE2
Lanthier et al^153^	2017	Decompensated alcoholic hepatitis	RCT	1	28/30	Autologous BM	Hepatic artery	4.7 × 10^7^	No improvement
Lin et al^148^	2017	ACLF associated HBV	RCT	6	56/54	Allogeneic BM	Peripheral vein	1.0 × 10^6^	MELD score, bilirubin, and survival rates
Detry et al^152^	2017	Liver transplant recipients	Non-RCT	12	10/9	Allogeneic BM	Peripheral vein	1.5-3.0 × 10^6^	No difference in the rate of infection or *de novo* cancer
Sakai et al^160^	2017	Liver cirrhosis—liver disease of mixed aetiologies	Cohort	12	4/0	Autologous Adipose	Hepatic artery	6.6 × 10^5^	Improvement of liver function, HGF, and IL-6 increased
Zhang et al^161^	2017	Ischemic-type biliary lesions following liver transplantation	RCT	24	12/70	Allogeneic UC	Peripheral vein	1 × 10^6^/kg, 6-time	Improved liver function and survival rate
Fang et al^142^	2018	Chronic HBV-associated liver cirrhosis	RCT	12	50/53	Allogeneic UC	Peripheral vein	(4.0-4.5) × 10^8^	MELD score, Child-Pugh score, and liver function
Casiraghi et al^150^	2021	Liver transplant recipients—liver disease of mixed aetiologies	RCT	12	9/10	Autologous BM	Peripheral vein	1-2 × 10^6^/kg	Safety of MSCs in liver transplant recipients. Increase in T reg cells and tolerant NK cells.
Schacher et al^149^	2021	CLD of mixed aetiologies (HCV/alcohol mainly)	RCT	3	4/5	Allogeneic BM	Peripheral vein	1 × 10^6^	MELD score, Child Pugh score and ACLF grade

Abbreviations: ACLF, acute-on-chronic liver failure; ALT, alanine aminotransferase; AST, aspartate aminotransferase; BM, bone marrow; CD4, cluster of differentiation 4; CLD, chronic liver disease; GGT, γ-glutamyltransferase; HBV, hepatitis B virus; HCV, hepatitis C virus; HGF, hepatocyte growth factor; IL, interleukin; IgM, immunoglobulin M; MELD, model for end-stage liver disease; MSC, mesenchymal stromal cell; NK, natural killer; PBC, primary biliary cholangitis; PGE, prostaglandin E2; RCT, randomised controlled trial; TGF, tumour growth factor; Th, T helper cells; TNF, tumour necrosis factor; T reg, regulatory T cells; UC, umbilical cord; UDCA, ursodeoxycholic acid.

### Role of MSC in Human Liver Cirrhosis

Liver cirrhosis is a consequence of chronic liver injury and is characterized by profound scarring and architectural disruption. Mohamadnejad et al^134^ used infusions of autologous BM-derived-MSCs in 4 patients with decompensated liver cirrhosis. Results confirmed that the approach was feasible and safe, and there was also an improvement in the Model for End-Stage Liver Disease score in 3 out of the four patients at 6 months.^135^ An initial case–control study by Amer et al^136^ included 20 patients with either chronic Hepatitis C or B-induced cirrhosis that received MSC either via the intra-hepatic or intra-splenic route. Compared to the control group, there was an improvement in, the MSC group’s MELD score, ascites, and peripheral edema.

Several other studies showed similar improvements in patients with viral-related cirrhosis,^137-139^ and 2 larger randomized-control trials (RCTs) by Salama and Xu et al,^140,141^ respectively, with a total patient number of 47, also demonstrated an improvement in MELD score, Child-Pugh score, in addition to reductions in IL-6, IL-17, and TNFα levels after MSC administration. A larger controlled trial that included 50 patients with chronic hepatitis B showed similar improvements. In the treated group, there was a marked reduction in IL-6 and TNFα levels. IL-10 increased at 2 and 4 weeks post-MSC transplantation. CD_4_T and Treg cells were higher in the treated group than in the control group at 2 and 4 weeks, whereas CD_8_T and B cells were markedly reduced.^142^ the Similar benefit was demonstrated in the largest phase 2 trial for patients with alcohol-related cirrhosis. Here 55 patients completed the study (19 in a one-dose MSC group, 19 in a 2-dose MSC group, and 18 in the control group) and were followed up for 12 months. The one-time and 2-time MSC groups showed a reduction in the proportionate collagen area following MSC therapy with figures of 25% and 37%, respectively; however, there was no difference in fibrosis between the groups.^143^

Finally, 2 cohort studies by Wang et al^144^ investigated MSC in the setting of UDCA-resistant PBC. Although these trials used small numbers (*n* = 17) of patients with no control group, results demonstrated a reduction in liver biochemistry, mainly alkaline phosphatase and gamma-glutamyl transferase.^144,145^ While initial findings from these studies are promising, many of these studies are small in number, short in duration, and often lacking a control arm. Larger and double-blinded controlled trials are required across different liver cirrhosis etiologies with a wider geographical distribution to reliably assess the role of MSC here.

### Role of MSC in Acute on Chronic Liver Failure

Acute on chronic liver failure (ACLF) is a condition characterized by systemic inflammation and organ failure and carries a poor prognosis. A case-control study by Peng et al^146^ included 53 patients with ACLF caused by Hepatitis B and 105 in the control arm group. The use of MSC demonstrated short-term efficacy but no effect on long-term outcomes. Hence albumin levels improved at 3-24 weeks post MSC infusion but not beyond that. Similarly, Bilirubin and PT levels improvement was limited to 4-12 weeks. The MELD score was superior to the control group at 3-36 weeks post-transplantation. There were no differences in mortality between the 2 groups over a 192-week period.^146^

Further studies by Shi et al^147^ and Lin et al^148^ demonstrated safety with MSC use and also an improvement in survival rates associated with an improvement in liver function and a reduction in the incidence of severe infection. In Lin et al’s paper, the rate of severe infection was 33.3% in the control arm receiving standard therapy versus 16.1% in the MSC group. Mortality rate from multi-organ failure was also lower in the MSC group—17.9% versus 37% in the control arm.^148^ Shi et al describe a significant improvement in PT levels over a 48-week period in those receiving MSC therapy versus the control group. This suggests the possibility of improved thrombin function in those receiving MSCs. After MSC infusion, the MELD score significantly decreased at 4, 8, and 12 weeks significantly decreased at 4, 8, and 12 weeks compared to the control group.^147^

The limitation in mortality benefit in Peng et al’s trial may be reflected in using a single autologous infusion of MSCs. The Shi et al and Lin et al studies used multiple allogenic infusions of MSCs over several weeks with an overall improvement in survival rates. However, autologous MSCs from patients with Hepatitis B may proliferate more slowly, and thus patients may require further transfusions to potentially improve efficacy.

In a more recent randomized placebo-controlled phases I-II trial, 4 patients with ACLF were treated with 5 infusions of BM-MSC for 3 weeks and compared to 5 patients in the control arm who received standard medical therapy and placebo saline infusion. This demonstrated the safety of MSCs; however, the 90-day survival rate was similar between both groups (20% for placebo versus 25% for BM-MSC).^149^

There are notable differences between the studies related to ACLF and its various etiologies. The earlier studies included only patients with Hepatitis B whilst Schacher et al’s recent study included patients with a wider range of etiologies (Hepatitis C and alcohol-related liver disease) and with more severe forms of ACLF—grades 2 and 3. This study was significantly underpowered with regard to mortality outcomes due to the small number in the intervention arm. Only one patient completed the infusion protocol due to the high ACLF mortality rates.^149^

### Role of MSC in Liver Transplantation

Two of the most common complications in patients post-transplant are rejection and infection. Casiraghi et al^150^ aimed to assess the safety profile of MSC in post-transplant patients using a single pre-transplant IV infusion of MSC given to 19 patients in total (10 controls). Over a 1-year follow-up period, there were no infusion-related complications post-MSC administration. Liver graft function remained similar in both groups. Immunologically, T reg and memory T regs increased for the first 2 weeks post MSC infusion; however, their levels at 6-12 months were comparable to the control arm.^150^

In a pilot study by Shi et al^151^, 14 patients with acute liver allograft rejection were given MSC, which improved liver function and graft histology. ALT, AST, and total Bilirubin significantly reduced in the 12-week follow-up period in those patients receiving MCSs. Histologically, portal vein endothelitis and bile duct damage improved in 42.8% of patients at 4 weeks post MSC infusion. However, these patients had not responded to immunosuppressive agent dose adjustments in relation to their acute rejection.^151^

In contrast, though Detry et al^152^ demonstrated that infusion of MSC in 10 liver transplant patients (with 10 controls) did not have any impact on the rejection rate or overall graft survival. A difference between these trials is that in the first trial, MSCs were given as a therapeutic option, while in the second trial, MSCs were given a few days after transplant with the aim of withdrawing standard immunosuppression. In Detry et al’s study, immunosuppression weaning was unsuccessful in the MSC group.

Mohamadnejad et al^134^ infused autologous BM-MSC in 15 patients with decompensated cirrhosis, with 12 patients in the control group. There were no changes in MELD score, Child-Pugh score, serum albumin, INR, and serum transaminases between the MSC and control groups over a 12-month follow-up period.^134^ This contrasts with Mohamadnejad’s initial study in 2009, which suggested an improvement in both liver function and quality of life in a similar cohort of patients, although the lack of a control group is a major limitation of the initial study.

Additionally, Lantheir et al^153^ treated 28 patients with alcohol-associated hepatitis with autologous BM-derived CD34+ stem cells and MSC. In the control arm, 30 patients received standard supportive therapy. Overall, there was no difference in hepatocyte proliferation on liver biopsy at 4 weeks in both arms. Macrophage activation in alcohol-associated hepatitis was reported to be a positive prognostic marker in a prior study by the same group. Although the trial displayed negative results, the greater level of macrophage activation in the MSC arm may warrant further study.^153^

Larger and more robust randomized clinical trials, both in the pre-clinical and clinical setting, are required to provide definitive evidence of MSC efficacy. In addition, such studies need to be undertaken across a more diverse geography to ascertain if the beneficial effects are consistently observed. Further work should also delineate the impact of MSC cell source, the best delivery route, and the importance of optimizing culture conditions of MSC.

### Utilizing MSCs and EV Therapy for Hepatic Disease

**Figure 3.**
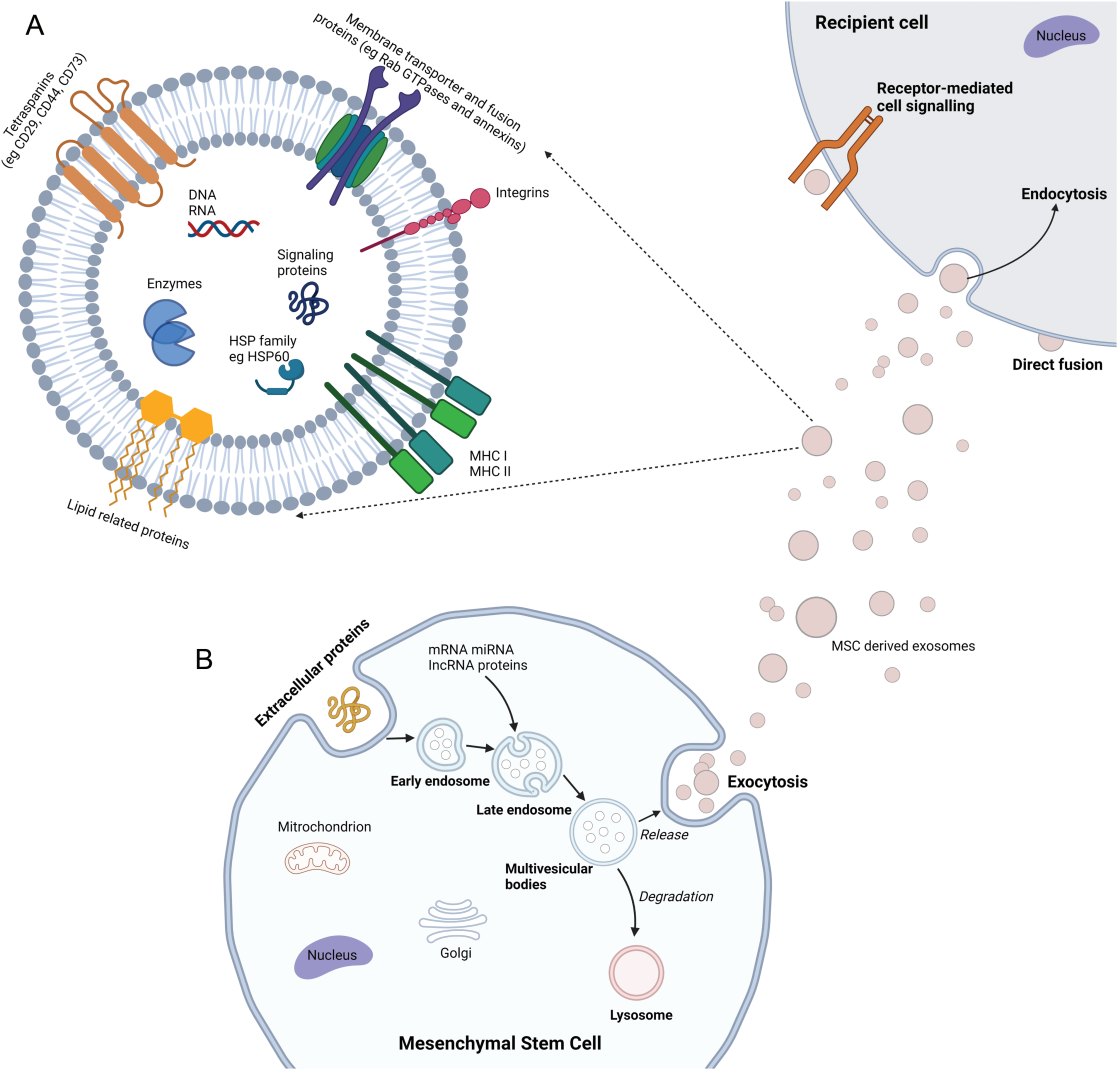
EVMs. (**A**) Extracellular proteins are invaginated via the cell wall to form early endosomes. Late endosomes develop into multivesicular bodies (MVB) via a selection of protein cargo. MVBs then either fuse with the plasma membrane resulting in exosome release or undergo degradation by lysosomes. The released exosomes then enter the recipient cell via 3 methods: receptor medicated cell signaling, endocytosis and direct cell fusion. (**B**) MSC-derived exosomes carry various proteins supporting its biogenesis—eg, proteins (eg, ALIX, TSG101), tetraspanins (eg, CD29, CD44, and CD73), membrane transporter and fusion proteins (eg, Rab GTPases and annexins), integrins, heat shock proteins (eg, HSP60, HSP70, and HSP90), and MHC classes I and II protein.

Various clinical trials thus far have investigated the role of MSCs in many liver diseases, the majority being in patients with Hepatitis B and C. Due to recent breakthroughs in antiviral therapy, Hepatitis C has become a curable disease; thus, the future role of MSCs in this cohort of patients is uncertain. In those with Hepatitis B, there are various effective antiviral therapies in development, which may deem it a curable disease soon. In other liver aetiologies, such as alcohol-related liver disease and NAFLD—MSCs have an anti-inflammatory and anti-fibrotic role. However, the exact anti-fibrotic mechanisms remain uncertain, although these have been postulated in the literature as described below (Fig. 3).

## Potential Risks of MSC Therapy

### Tumourigenic

Despite numerous studies reporting on the safety profile of MSCs, there have been contradictory results describing the pro and anti-tumorigenic effects of MSCs. One potential concern is the susceptibility of MSCs to undergo malignant transformation, as MSCs undergo several passages before transplantation, potentially increasing their risk for genetic mutations, both in vitro and in vivo. Murine MSCs exhibited chromosomal changes and transformation into malignant cells.^162-166^ Paradoxically, several studies demonstrated human MSCs becoming senescent both in vitro and in vivo.^163,167,168^ Dahl et al^169^ showed that a high passages N170, MSCs may display telomeric deletions, and a further study observed microsatellite instability and downregulation of genes involved in DNA repair in human MSCs.^170^

MSCs have a potential risk of promoting pre-existing tumor growth or precancerous lesions, and in vitro, when transplanted with cancer cells, MSCs have been shown to promote tumor formation by releasing various growth factors.^171^ In contrast, Cousin et al^172^ highlighted tumor growth inhibition in pancreatic cancer cells in vivo. In animal models, MSCs have shown conflicting results—both promoting^173-176^ and inhibiting tumor growth.^177-179^

Casirgaghi et al^180^ published a safety study in 700 patients demonstrating that no patients developed any tumors from autologous or allogeneic MSCs. This would suggest that there are fundamental differences between human and murine MSC when it comes to the risk of tumorigenicity, as human MSC have not been associated with any such events.

### Viral Transmission

Autologous MSC transplantation may carry a risk of viral transmission to the recipient, as has been demonstrated in the treatment of GvHD after hemapoietic stem cell transplantation.^138,181^ Sundin et al^182^ demonstrated that MSCs can be infected with CMV and HSV-1 in vitro, but not EBV, although small subpopulations of MSC expressed CD21—receptor for EBV uptake in those patients. On the other hand, parvovirus B19 transmission to bone marrow cells in vitro carries a low pathogenicity, and patients transplanted with allogeneic B19-positive MSCs did not result in symptom development or viraemia.^182^

MSCs are known to reduce lymphocyte responses to bacteria, fungi, and viruses, but it’s relevance needs to be evaluated further in clinical trials . As yet, no information is available on the role of HSV and CMV transmission in vivo, but to reduce the risk of viral transmission, both recipient and MSC donors should be screened for the above viruses, as they can be fatal in immunocompromised patients.

## Bridging the Gap Between Optimism and Reality

Despite the encouraging reports from murine studies and early phase clinical trials, there remain a series of challenges and issues that need to be addressed before MSC can be translated into meaningful therapeutic products for patients.

### Improving Lab-Based Mechanistic Cell Therapy: From Bench to Clinic

Animal models often inadequately reflect human disease, which is compounded by species-specific differences in the immunobiology of human and murine MSC. Thus, according to Ritskes-Hoitinga et al,^183^ specific measures have been suggested to create in vivo systems that replicate native human tissues for cell testing to overcome the limitations of in vivo models.^184^ In this way, Hu et al^185^ illustrated that the creation of human liver disease models for use as test platforms has been made possible by using human-induced pluripotent stem cell (hiPSC) technology. When sufficient evidence demonstrates the predictive value of these models for efficacy and toxicity data, alongside well-established and validated relevant models for each disease, it will hopefully be possible to replace animal testing with human 3D in vitro liver tissue models. Humanized mouse systems can be utilized, and Bissig-Choisat et al^186^ conducted research using a unique NAFLD humanised mouse system, with metabolic analyses of humanized livers demonstrating similarities to those seen in people with liver disease.^187^

### MSC Product Optimization

Potent effects of MSC therapy may only be observed in 45%-50% of the patients,^188,189^ which is not dissimilar to the effects observed with other cellular therapy products in development. Suboptimal clinical response is dependent on a variety of variables, including heterogeneity of the MSC, patient recipient disease heterogeneity, variable dosing, and dosing routes, and limited understanding of the host response.^189,190^ Inherent heterogeneity between sources of MSC is due to multiple factors including donor characteristics, tissue origin,^191^ and also the isolation and in vitro preparation methods.^192,193^ This point can be illustrated by the results of the phase III trial conducted for graft versus host disease (GVHD) using prochymal (a commercial MSC product).^194^ MSC used in this trial were retrieved from a single donor, expanded to passages 3 and 4 during manufacturing, and infused at 2 million cells/kg twice a week for 4 weeks to treat 240 participants. The trial did not meet its primary clinical endpoint of complete response in GVHD in comparison to placebo control, although a positive impact was noted on the gut and liver. However, these findings were in contrast to published European data,^195^ where a phase II trial^138^ to treat a similar patient population with GVHD demonstrated clinical efficacy with a response rate of 77% in patients with GVHD at the primary endpoint (day 28). In this study, MSCs were pooled from 8 donors, and variable doses (median 2.2 × 10^6^ cell/kg) with a median of 3 doses (range 1-9) were administered. These examples highlight the need for developing standardized protocols for MSC production to allow meaningful comparisons between different studies. In addition, they suggest that careful identification of potency markers in MSC batches made from different donors is important to maximize efficacy.^152^

### Priming of MSC

To cater for diverse immune disorders and to augment their therapeutic efficacy, priming and purification of MSC may be required^196^ as there are marked differences in gene expression of therapeutically effective and ineffective MSC clones, as demonstrated by Lee et al^197^ Among the genes expressed by effective clones, endothelin-1(EDN-1) significantly increased the efficacy of human UC-MSC against myocardial infarction. In addition, mechanistic analysis of EDN-1 showed significantly increased expression of Cadherin2 (CDH2) and vascular endothelial growth factor (VGEF). To improve the predictability of MSC effector functions, the Clinical Indications Prediction (CLIP) scale has been developed^198^ to predict the impact of culture conditions and donor to donor heterogeneity on the therapeutic efficacy of MSC for different disease indications.

Priming MSC in vitro with stimulatory factors is another strategy to improve their engraftment and therapeutic functions, albeit complicated by cost of goods considerations. Agents tested to date include the use of cytokines or growth factors such as TNF-γ, IL-β1, TNFα, IL-17, TGFβ-1^199-202^ as well as hypoxic culture conditions,^135,203^ modification of culture methods^204^ and genetic modification of MSC^205^ to improve potency.

Twist1 has been demonstrated to be a direct target of fibroblast growth factor (Fgf2) and interferon-gamma in MSCs, thus allowing an opportunity to alter the immunomodulatory and anti-inflammatory effects of MSCs.^198,206-208^ Bauer et al^209^ developed an in vitro assay that integrated multidimensional flow cytometry data into a measurement of MSC-mediated inhibition of T-cell activation. They identified distinct morphological subpopulations that could be predictive of MSC immunosuppressive function via activation of CD4+ and CD8+ T cells. In addition, HOXB7 overexpression was found to potentially increase MSC proliferation by inducing a fibroblast growth factor-mediated autocrine loop.^210^

MSCs are thawed and directly infused as the standard of care in most clinical studies. Notably, during cryopreservation, the actin filaments within the MSCs are disrupted resulting in death in the bloodstream prior to reaching their target destination.^211^ Nolta et al^211^ demonstrated that MSCs prepared with cryo-recovery and hypoxic formulation pre-infusion have better survival than unconditioned cells in immune-deficient mice (10% versus <1% in pre-conditioning). To improve in vivo survival and preserve MSC function, there needs to be a period of recovery after cryopreservation prior to injection. Reducing sugars in the post-thawing period has also been shown to improve the duration of MSC retention and functionality in vivo revascularization assays.^212^ Courtman et al’s phase I trial on MSCs in septic shock used freshly infused allogeneic bone marrow-derived MSCs, which were demonstrated to be safe (www.clinicaltrials.gov; NCT02421484).

In a study of ARDS, MSC direct coculture with monocyte-derived macrophages enhanced their phagocytotic activity.^213^ In a separate study by Le Blanc et al,^214^ in patients with steroid-resistant severe GvHD, 71% of patients responded to MSC therapy but with varying survival rates. Follow-up data suggested that “”responders’’ had a gut immune profile with higher levels of CD8+ lymphocytes and FoxP3+ T lymphocytes and lower levels of CD56+ and CD68+ compared to “”non-responders’’. This suggests that the ongoing gut inflammation in recipients may affect the therapeutic potential of MSCs. Further studies to explore the role of a patient’s immunogenic profile and their effect on MSCs.

### Route of Administration

There is no consensus yet on the best delivery route for MSCs for clinical studies relating to liver disease, with the peripheral vein being the most widely used, followed by the hepatic artery. Some trials used more directed methods via the intra-splenic and intra-hepatic routes, but there was no difference in efficacy based on the route of administration (PV, IS, portal vein, or IH).^136,155^ injection could lead to cell damage by trauma, hypoxia, or NK cell-mediated MSC apoptosis, as well as adding logistical and financial consequences. In contrast, MSC systemic administration may limit biodistribution and homing effects on the target tissue.

MSC delivery has been demonstrated to trigger a prothrombotic state via activation of the complement system and coagulation cascade, known as “Instant Blood-Mediated Inflammatory Reaction” IBMIR, although thrombotic events have been reported in only a few studies.^215,216^ MSC infusion increases C3a and sC5b-9 levels, activating the thrombin anti-thrombin complex, resulting in a pro-coagulant state.^217^ Further studies are required to establish the optimum route of delivery that would improve the efficacy of MSC-based therapies.

Other clinical challenges to address include standardizing dosing regimens (single versus multiple doses) and cell therapy release assays as relevant for diseases and differing patient populations.^189^ Various dosing regimens have been used in clinical trials and given that most of these trials have been aimed at determining the efficacy, an optimal dose of MSC for clinical use has not been established.^218,219^ For example, in a study using MSC in liver cirrhosis, MSCs were injected at a dose of 1 × 10^7,^ and they were found to be effective for 6 months.^139^ In the same year, 2013, another study with MSC administration in liver cirrhosis found a dose of 2 × 10^8^ to have no significant effect after 12 months compared with a placebo.^134^ Thus, heterogeneity between studies poses challenges in comparing the different trials.

### Analytical Potency Methods

The International Society for Cellular Therapy (ISCT) identified the need for functional markers of potency and the development of release potency assays to meet regulatory requirements for advanced clinical trials.^220^ Three preferred analytical methods were suggested: quantitative RNA analysis of selected gene products, flow cytometry analysis of functionally relevant surface markers, and a protein-based assay of the secretome. MSC poses a certain challenge to potency analytical methods development due to the various tissue sources, heterogeneity of proposed mechanisms, and lack of reference standards. To date, only a few potency assays have been studied—robust and reproducible quantitative potency assays for MSCs are needed to accelerate the transition into clinical practice. In addition, these assays will need to be validated for analytical procedure, specificity, accuracy, and precision.

In order to improve MSC safety in those with hepatic disorders, we need to be selective in choosing the relevant patient population, ensuring patients have the right indication. Alongside this, we need to ensure that the appropriate donors are selected and that the MSCs undergo a rigorous handling process in terms of cryopreservation, storing, and thawing. The optimum delivery of MSCs in hepatic disease is yet to be defined. To reduce the risk of immediate reactions following MSC infusion, pre-medications such as steroids and antihistamines may be considered.

## Conclusions

The data available thus far provides a strong foundation to robustly investigate the potential of the MSCs and overcome the remaining challenges before MSC can be used as a clinical treatment. In comparison to MSC, MSC-derived EVs, with their higher safety profile, lower immunogenicity, and safer cargo of EV contents between recipient and donor cells, also warrants further research to harness their full potential. Integrated efforts between scientific, regulatory, industrial, and clinical stakeholders to expedite the production of MSC that are optimized and tailored for the indication of use and readily available at an affordable cost are required to make a meaningful progress.

## Data Availability

There are no new data associated with this article. DOI links to the translational and clinical studies appraised in this review are cited in the references section.
